# Every breath counts: Lessons learned in developing a training NICU in Northern Tanzania

**DOI:** 10.3389/fped.2022.958628

**Published:** 2022-08-25

**Authors:** Stephen J. Swanson, Kendra K. Martinez, Henna A. Shaikh, Godbless M. Philipo, Jarian Martinez, Evelyine J. Mushi

**Affiliations:** ^1^Department of Paediatrics, Arusha Lutheran Medical Centre, Arusha, Tanzania; ^2^Global Pediatrics Program, University of Minnesota, Minneapolis, MN, United States; ^3^Global Health Center, Children's Hospital of Philadelphia, Philadelphia, PA, United States; ^4^Department of Paediatrics and Child Health, Tygerberg Hospital, Stellenbosch University, Cape Town, South Africa

**Keywords:** neonatal intensive care unit (NICU), physician training, prematurity, kangaroo care (kc), global neonatology, Tanzania, Sub-Saharan Africa, neonatal mortality (NM)

## Abstract

**Introduction:**

Neonatal mortality rates in resource-limited hospitals of Sub-Saharan Africa (SSA) remain disproportionately high and are likely underestimated due to misclassification of extremely preterm births as “stillbirths” or “abortions”, incomplete death registries, fear of repercussions from hospital and governmental authorities, unrecorded village deaths, and cultural beliefs surrounding the viability of premature newborns. While neonatology partnerships exist between high income countries and hospitals in SSA, efforts have largely been directed toward improving newborn survival through neonatal resuscitation training and provision of equipment to nascent neonatal intensive care units (NICUs). These measures are incomplete and fail to address the challenges which NICUs routinely face in low-resource settings. We draw on lessons learned in the development of a low-technology referral NICU in Tanzania that achieved an overall 92% survival rate among infants.

**Lessons learned:**

Achieving high survival rates among critically ill and preterm neonates in SSA is possible without use of expensive, advanced-skill technologies like mechanical ventilators. Evidence-based protocols adapted to low-resource hospitals, mentorship of nurses and physicians, changes in hierarchal culture, improved nurse-infant staffing ratios, involvement of mothers, improved procurement of consumables and medications, and bedside diagnostics are necessary steps to achieving high survival rates. Our NICU experience indicates that low-technology solutions of thermoregulation, respiratory support *via* continuous positive airway pressure, feeding protocols and infection control measures can ensure that infants not only survive, but thrive.

**Conclusions:**

Neonatal mortality and survival of preterm newborns can be improved through a long-term commitment to training NICU staff, strengthening basic neonatal care practices, contextually appropriate protocols, and limited technology.

## Introduction

Neonatal mortality now comprises approximately half of under-5 mortality worldwide (2.4 million out of 5 million yearly deaths) with the highest burden in Sub-Saharan Africa (SSA). Within the first month of life, prematurity (36%) and birth asphyxia (24%) remain the leading causes of death (1, 2). Moreover, there are an estimated 2 million stillbirths each year and 40% of these are thought to be due to preventable intrapartum events (3). Combining these, neonatal and perinatal deaths surpass 3.2 million each year, with 1.4 million deaths (43%) occurring in SSA (1–3). Not only are stillbirths (including fresh stillbirths) not included in overall mortality data, but many neonatal deaths and stillbirths are neither appropriately recorded nor issued a death certificate—suggesting that current neonatal mortality statistics underestimate the magnitude of the problem (4). In Tanzania, the neonatal mortality ratio (NMR) in 2020 remains high at 20 per 1,000 livebirths (90% uncertainty interval 14–29) (1). Globally, neonatal deaths account for 3% of annual mortality (all ages, both genders), but in Tanzania neonatal deaths comprise 11% of all-age mortality and 33.5% of under-5 mortality (5).

With over 1.3 billion people, Africa remains the second most populous continent with a 3.4% growth rate (6). Wide disparities exist within and between countries in the availability of specialists, sub-specialists, and trained neonatal nurses. A recent survey of 49 African countries indicated that 12 countries had no neonatologists, 21 countries had <50 pediatricians, and specialty neonatal nursing care was recognized in only 57% of surveyed countries (7). Any improvement in global neonatal outcomes requires rapidly increasing the implementation of evidence-based maternal and neonatal care in hospitals throughout low- and middle-income countries (LMICs) (8). Yet, many barriers exist including staffing constraints, cultural beliefs that extremely low birthweight (ELBW, <1,000 g) infants cannot survive, lack of neonatal knowledge and skillsets, poor provision of neonatal respiratory care, lack of appropriate medical equipment and medications, and competing healthcare priorities within health systems (7, 9–11). In this article we will share our journey in the development of an East African neonatal intensive care unit (NICU) and the subsequent effect on neonatal mortality.

## A NICU's journey in Northern Tanzania

### The early years

In 2013, Arusha Lutheran Medical Center (ALMC) was in its fifth operational year, a 145-bed hospital offering care for ~10,000 inpatients (children and adults) and 108,000 outpatients annually and with dramatic year-over-year patient growth. Twenty-eight physicians (13 specialists) and 136 nurses were on staff, including two obstetricians and two pediatricians. Like many African hospitals, ALMC relied largely on junior doctors (interns and medical officers) to see most patients and experienced a 10% turnover of staff each year. Furthermore, it's “NICU” consisted of a single, ill-equipped room adjacent to the obstetric ward where unstable newborns were placed—a unit added as an afterthought for babies too ill for the post-partum ward. Oxygen delivery was limited and donated equipment (e.g., radiant warmers, incubators, and phototherapy lights) frequently malfunctioned and lacked replacement parts. Necessary equipment, consumables, and essential medications were largely absent or dependent on inconsistent international donations. Infants in respiratory distress relied on makeshift bubble continuous positive airway pressure (bCPAP) setups utilizing 100% FiO_2_.

Compounding this was a significant shortage of Tanzanian nurses and doctors qualified to work in a NICU. Nurses were assigned 6–8 infants and rotated to a different hospital department (medicine, surgery, pediatrics, labor ward, and clinics) every 3 months—a widespread nursing practice still employed in many African hospitals based on a conviction that nurses (and doctors) should be able to care for all patients with equal competence. Because of this, nurses oriented to the NICU routinely failed to achieve necessary procedural skills or familiarity with common newborn conditions. Interns and the medical officer (“registrar”) responsible for NICU patients similarly lacked formal training in either pediatrics or neonatology. Few written protocols existed to guide medical decisions with contextual relevance for an African hospital. With these staffing challenges and knowledge gaps, a baby's deterioration in the NICU often went unnoticed until little could be done.

Not surprisingly, our overall NICU mortality rate was 19–24% during 2013–2014 (Table 1). For ELBW infants, the mortality rate was 80% (Table 2) and extremely preterm infants rarely survived. ALMC, however, was not a unique story and faced similar challenges to many hospitals across SSA—where NICU outcomes appear deceptively better due to classification of live births ≤28 weeks gestation as “abortions” or “stillbirths”.

**Table 1:** NICU total admissions, prematurity, referrals, surgical admissions, and adjusted survival by year from 2013 to 2021.

	**2013**	**2014**	**2015**	**2016**	**2017**	**2018**	**2019**	**2020**	**2021**
Total admits (YoY % Δ)	117 (-)	222 (90%)	236 (6%)	209 (−11%)	251 (20%)	238 (−5%)	316 (33%)	325 (3%)	340 (5%)
Premature (% of total)	49 (42%)	75 (34%)	74 (31%)	104 (50%)	84 (33%)	74 (31%)	129 (41%)	144 (44%)	175 (51%)
Outborn referrals (% of total)	25 (21%)	111 (50%)	119 (50%)	95 (45%)	87 (35%)	60 (25%)	149 (47%)	130 (40%)	169 (50%)
Surgical (% of total)	11 (9%)	18 (8%)	20 (8%)	14 (7%)	9 (4%)	14 (6%)	22 (7%)	28 (9%)	29 (9%)
Gross survival rate	81%	76%	80%	82%	90%	88%	90%	87%	87%
Adjusted survival rate	82%	77%	81%	83%	90%	93%	92%	93%	92%

Adjusted survival (%) excludes admitted newborns with congenital cardiac defects, gastrointestinal or major birth anomalies, or preterm neonates ≤25 weeks' gestation, as these newborns are unable to survive in most NICU settings in SSA. YoY, Year-over-Year.

**Table 2:** NICU admission and survival rates by birthweight categories and interventions by year 2013–2021.

	**2013**	**2014**	**2015**	**2016**	**2017**	**2018**	**2019**	**2020**	**2021**
Total admits	117	222	236	209	251	238	316	325	340
<1,000 g ELBW admissions (survival rate)	–	10 (20%)	14 (29%)	20 (45%)	14 (64%)	10 (33%)	13 (73%)	22 (79%)	32 (74%)
1,000–1,499 g admissions (survival rate)	–	27 (67%)	16 (56%)	35 (71%)	21 (71%)	24 (74%)	35 (91%)	44 (83%)	59 (85%)
1,500–2,499 g admissions (survival rate)	–	58 (90%)	59 (89%)	47 (96%)	69 (97%)	48 (93%)	82 (97%)	86 (91%)	78 (99%)
**Interventions**
bCPAP (% of total)	22 (19%)	69 (31%)	79 (33%)	74 (35%)	81 (32%)	72 (30%)	180 (57%)	207 (64%)	228 (67%)
Pulmonary surfactant (% of total)	N/A	N/A	N/A	N/A	22 (9%)	27 (11%)	47 (15%)	58 (18%)	65 (19%)
Phototherapy (% of total)	40 (34%)	95 (43%)	92 (39%)	110 (53%)	122 (49%)	111 (47%)	164 (52%)	179 (55%)	194 (57%)

(-) data not recorded or incomplete. Pulmonary surfactant widely unavailable in Tanzania prior to 2017. (N/A) Pulmonary surfactant widely was unregistered and widely unavailable in Tanzania prior to 2017.

### Building a NICU starts with nurses

In late 2014, we simultaneously undertook many steps to begin improving the NICU, including expanding the unit and removing non-functional equipment. One of the most foundational steps involved redefining the role of nurses. With support from nursing leadership, mandatory nurse rotations ended, yielding a more stable nursing team. We partnered with experienced volunteer NICU nurses from the USA and Canada to build skills and knowledge among our nurses. Short-term volunteer stints were highly discouraged unless the nurse instructor had previous experience and established relationships with our NICU staff. On average, clinical nurse instructors came for 4 continuous months (range: 6 weeks−2 years), allowing time to learn our hospital system, strengths and limitations, cultural differences and, most importantly, build trust and comradery with Tanzanian nurses. Visiting nurse instructors partnered with Tanzanian nurses in an accompaniment role without removing work duties from local nurses.

Basics of nursing care were prioritized, including thermoregulation, non-invasive respiratory support, neurodevelopmental positioning, feeding and nutrition, infection control measures, and safe delivery of medications. Tanzanian nurses were assigned to care for specific infants to increase accountability and continuity of care. Patient assignments became an accepted new approach, as “everybody's baby is nobody's baby”. Use of nursing assessments were encouraged, and intentional efforts were made to involve nurses in daily rounds and clinical decisions. This worked to reduce the preexisting physician-nurse divide and emphasize the value of nursing judgment. Nurses were encouraged to speak to physicians when concerned about the status of a baby or unclear about a medication or plan. In time, the NICU became known as a supportive environment and greater numbers of nurses wanted to work there, and a 1:3 nurse-to-baby staffing ratio was able to be achieved (many large SSA hospitals continue to staff at 1 nurse per 20+ NICU infants).

### Accompanying doctors

Another important step was the implementation of physician mentorship to promote deeper knowledge and skills specific to neonatology. According to estimates, there are only 0.23 doctors per 1,000 people living in SSA with an urgent need to educate more doctors (12, 13). Nonetheless, most literature on global health education focuses on learners from high-income countries (HIC)s who spend time in LMICs. In our experience, training doctors is a long-term process of accompaniment. Below, we list key tenets that have served us as we have accompanied doctors in this process.

First, we reduced dysfunctional hierarchies and encouraged team members at all levels to speak up. In Tanzania, the legacy of colonialism mixed with a cultural respect for elders and the need to uphold the ideal of community promotes a strict hierarchy whereby learners, junior doctors, and nurses rarely challenge their superiors. Yet we know patient outcomes improve when medical teams function cohesively and all members are encouraged to speak up (14, 15). This can be modeled in the unit as we navigate diagnostic uncertainty and cognitive biases with humility by readily welcoming challenges as learning opportunities and openly valuing others' opinions.

Second, we created safe learning environments. There must be adequate and supportive supervision so that trainees can make mistakes without harming patients. There is no room for shaming when promoting growth (16, 17). Doctors should never be punished nor humiliated for making unintended mistakes, and they should be celebrated for acknowledging their learning gaps and asking questions. In other words, we granted doctors the “permission not to know” and provided ample educational resources.

Finally, we encouraged critical thinking and attention to detail rather than regurgitating memorized facts. We can help doctors develop skills for synthesizing clinical information into a coherent assessment so they can formulate diagnostic and management plans while accounting for clinical uncertainty and personal cognitive biases (18). We can also emphasize the importance of details, especially when working with newborns, as small changes can make a huge difference. These skills equip learners to continue refining their capacity to manage sick newborns even after we leave.

While short-term education trips and virtual trainings have their utility, the tenets we list above are best taught at the bedside through long-term, in-person accompaniment. Through this model we have seen four former junior physicians (registrars) from ALMC go on to become consultant pediatricians in Tanzania, with more registrars currently pursuing or soon to begin their pediatric residency training.

### Partnering with families

Attention was also given to the NICU parents and caregivers who were encouraged to actively participate in daily cares and share their concerns regarding their infant with the medical team. Despite not having sufficient space for an appropriately sized kangaroo care unit, parents routinely employ skin-to-skin (kangaroo care) at every opportunity in the NICU. Families are also taught how to fortify expressed breastmilk using locally available formula and use of a secured feeding tube to feed their preterm infant or infant on bCPAP. As communication between parents and providers increased, we gradually shifted away from the physician-centric model of care.

### Judicious use of technology

The use of technology in our NICU was intentionally kept simple. NICUs in LMICs operate in a very different context and serve to meet different needs from those in HICs (19, 20). In HICs, systems for addressing basic newborn care (e.g., newborn resuscitation, infection prevention and control, nutritional support, etc.) are relatively strong, so therapies to improve survival focus on increasingly technologically sophisticated solutions (e.g., conventional and oscillator ventilators, therapeutic cooling, ECMO, etc.). HICs also have many well-trained staff to install and maintain complex equipment. Contrastingly, problems like hypothermia, infection, hyperbilirubinemia, poor nutrition, and respiratory distress among moderately to late preterm infants remain rampant in LMICs (21–23). These problems are better addressed by carefully applying simple concepts and technologies.

Introducing complex technologies into a setting that is not ready to receive them can lead to more harm than good. There are many treatable neonatal conditions that do not rely on complex technologies and should be employed to save newborns in LMICs. In Africa, wide disparities persist between and within countries in the availability of basic, low-technology neonatal respiratory care (i.e., access to nasal prongs, use of high flow heated and humidified oxygen, surfactant, and CPAP). For example, 74% of surveyed African countries reporting that CPAP was available in <10% of cities with a population of more than 150,000 (7). Lifesaving, effective, low-cost technologies remain both inequitably distributed and poorly adopted in many hospitals today.

At ALMC, no ventilators are used in the NICU. Instead, we go back to the basics: thermoregulation, non-invasive respiratory support, optimizing nutrition, and infection prevention/treatment. In 2021, 228 of 340 infants (67%) in the NICU were treated with bCPAP (Table 2) which significantly improves survival rates in LMICs (24). Importantly, we are careful to use warmed, humidified air and oxygen blenders while also performing meticulous nasal care and therapeutic infant positioning. For infants with severe respiratory distress, we pair this with judicious and timely administration of pulmonary surfactant (often administered through a laryngeal mask airway) (25). Additionally, we are elevating our reliance on kangaroo care, which reduces both hypothermia and rates of infection (26). We've also prioritized infection prevention, including intravenous line care and minimizing shared medications and IV fluids. Through investment in a point-of-care EuroLyser Cube^1^ assay, which measures quantitative values of C-reactive protein (CRP) from a drop of whole blood, we can trend a non-specific marker of inflammation and better balance the need for early antibiotic treatment for suspected sepsis with antibiotic stewardship (27). Our focus is on early introduction of enteral feeds, rapid feeding advancement with breastmilk fortification, and sodium monitoring/replacement therapy—measures to improve nutrition and achieve optimal weight gain, as nutrition remains the best way to help neonates help themselves. Lastly, we've tackled simple pathologies like hyperbilirubinemia by using a transcutaneous bilirubinometer to detect dangerous levels of hyperbilirubinemia and promptly treat with phototherapy (23). While we've invested in some technologies, our primary focus remains investing in physicians and nurses who can recognize infants' needs and continue to build out a sustainable NICU going forward. Our 92% survival rates suggests that survival of preterm and critically ill term babies in Tanzania is possible.

Many studies have shown that therapies developed for HICs do not save lives, and may even lead to greater mortality, when used in LMICs. This was demonstrated in the HELIX trial of therapeutic hypothermia for asphyxiated neonates as well as a recent trial which found greater mortality among sick neonates cared for in incubators compared to kangaroo care (26, 28). Newborns in LMICs undeniably deserve the same caliber of care as newborns in HICs; meeting this goal requires first building strong foundational care practices before utilizing more advanced technologies.

Moreover, a recent review found that in SSA, while up to 70% of medical equipment is donated, only 10–30% of this equipment remains operational (29). In the current state, opportunities for failure arise throughout the supply chain of donated medical goods. An inherent power imbalance between donating and receiving parties, along with a lack of collaborative planning often leads to a mismatch between what equipment is needed and what is donated. Once it arrives in the recipient country, equipment designed for HIC can quickly become damaged when exposed to high temperatures and humidity, dust, and fluctuating electricity voltages common in LMIC. Once damaged, equipment is difficult to repair, since trained biomedical engineers and technicians are rare and spare parts often need to be imported—an expensive and time-consuming process (30). Furthermore, NICUs in LMICs often receive equipment from various manufacturers—each with their own upkeep, repair protocols, and spare parts (29). This leads to so-called “equipment graveyards”, where equipment lies defunct, simply taking up valuable space (19, 20, 31).

Efforts to ameliorate these challenges are ongoing. There are calls for greater partnership between donating and receiving institutions so that donated goods better meet local needs. Some non-governmental organizations (NGOs) and distributors, including NEST360^2^ and Kenya-based HATCH Technologies^3^, respectively, are attempting to provide appropriately durable equipment with biomedical engineering support for sustained use. Finally, ongoing efforts to support local innovation can help meet technological needs and foster healthcare system independence (19).

Saving newborn lives in LMICs requires looking beyond technologies used in HICs. Respiratory support of the NICU baby through conventional mechanical ventilation or high frequency oscillatory ventilation does not exist anywhere in northern Tanzania. Our training workshops consistently addresses questions by local healthcare authorities on whether a mechanical ventilator should be a prerequisite piece of equipment for a unit to call itself a “NICU” —reflecting a widespread belief that technology is what separates LMIC from HIC provision of care. Not surprisingly, our own past NICU history and outreaches to other hospitals suggest that neonatal deaths occur among babies due to aggressive and improper use of mechanical ventilation, particularly in settings where nurses and physicians are poorly trained and supportive measures (i.e., proper suction, portable x-rays, and blood gases) are lacking. Reliance on the possibility of mechanical ventilation (“if the baby deteriorates, we can put him on the ventilator”) may lead to neglect of a baby who might otherwise survive through prompt use of blended, warmed, and humidified CPAP, timely administration of pulmonary surfactant, and anticipatory nursing care. Use of mechanical ventilation in the NICU further diverts limited nursing care away from CPAP babies to the highest acuity, now-ventilated NICU baby. Multiple nurses may become bystanders, watching the baby on the ventilator at the neglect of other important nursing duties. This was our first-hand experience before we “retired” our ventilator due to space constraints and poorer outcomes. As such, we make every effort to help a baby survive through non-invasive respiratory measures.

### ALMC NICU today

From 2013–2021, our NICU annual census increased from 117 to 340 admissions, representing an annual growth rate of 14% over 8 years (Table 1). Gross survival rates have been 87–90% since 2018, and adjusted survival rates are ≥92% (Table 1). At present, ALMC does not have any mechanical ventilators in the unit, and yet in 2021 we were able to achieve an overall 90% preterm survival rate, with 74% and 85% survival rates for infants weighing <1000 grams and 1000–1499 grams, respectively (Table 2). This stands in contrast to the initial years of 2014-2015 when our survival rates for ELBW infants was 20-29% and 1000–1499 gram infant survival was 56–67% (Table 2). Currently, most of our very low birth weight (VLBW, <1500 grams) infant deaths occurs among transfers from referring private or public hospitals (or home deliveries presenting to the emergency department), where initial post-delivery stabilization is often lacking, and arrival comes too late. In 2021, 51% of all ALMC NICU admissions were preterm neonates and 50% of all admissions were outborn births. High-risk obstetric patients and ELBW/VLBW babies are now routinely referred to our hospital because of the presence of an established NICU. In 2021–22, our smallest survivor was 24 weeks gestation, and smallest recorded birthweight was 612 grams.

### Challenges and opportunities

Continued growth has been limited by lack of physical NICU and kangaroo care bed space as well as nursing shortages worsened by problems with nursing retention. Training a nurse to become a specialized NICU nurse is a lengthy process involving many months of supervision, and continued nursing turnover with delays in hiring replacement nurses compromise NICU outcomes and increase nurse: patient ratios. Procurement of needed NICU consumables and essential medications also remains difficult in our local setting, as needed supplies are unavailable through distributors in Tanzania. Some essential NICU drugs (i.e., caffeine citrate) remain unregistered in the country, despite being endorsed in both national Tanzanian and WHO guidelines. Acquisition and importation of needed supplies and equipment for daily operation remains subject bureaucratic obstacles and added clearance fees. Other supplies and medications for NICU babies are increasingly priced above what many families can afford. Striving for long-term sustainability remains hampered by competing hospital priorities, which limits reinvestment of generated revenues back into the NICU. All the while, we continually seek to make improvements sustainable and access equitable. To date, ALMC NICU has never turned away an infant due to payment issues with the belief that every baby deserves a chance live and thrive, regardless of family income and location of birth. Funding for our NICU initiative is often achieved through local fundraising efforts and international small donors when internal revenue falls short.

In 2021, we published our NICU protocol manual, “Every Breath Counts: Manual of Neonatal Care and Drug Doses” written for East African hospitals (32). This manual, along with a paired conference in which 55 hospitals participated (Tiny Feet, Big Steps: Advancing Care of Critically Ill and Premature Babies in Tanzania), created a venue to advance neonatology education and offer resources written in an East African hospital, for an African setting. Our NICU continues to engage local and national government officials through sharing of our neonatal outcomes and protocols, leading workshops in neonatology in both public and private hospitals, and hosting the annual Tiny Feet, Big Steps neonatology conference. Public and private hospital training of nurses and doctors in neonatology has been part of our earliest mission. We have established a national reputation for our training workshops, conferences, written protocols and manual, and the consistent demonstration of what can be achieved in a low-resource, low-technology NICU setting in Tanzania.

## Discussion

We remain committed to the ideals that public-private partnerships and NGOs can work to improve neonatal outcomes in LMICs. However, an approach that moves beyond helping babies survive toward helping NICU infants thrive must be adopted. Teaching and equipment must be accompanied with intentional efforts to build a culture where nurses are elevated, anticipatory and proactive approaches rewarded, and an expectation of a future healthy life exists for hospitalized infants. Training both nurses and doctors is a journey of accompaniment where team hierarchy is minimized, and safe learning environments with daily patient assessments are encouraged. Physician and nurse champions of the NICU must mentored and valued by hospital administration. Protocols and NICU guidelines need to be written that are both evidence-based and adapted for contexts where medications and diagnostics may be limited. Widely held beliefs that idealize equipment and promote the need of technology to catapult a LMIC hospital into sudden HIC status must be challenged. There are no shortcuts to developing a NICU that can achieve a survival rate of ≥92%. Our outcomes have reflected consistent investment in training and proper staffing levels of motivated NICU nurses and doctors, building a culture of respectful teamwork, and consistent focus on the basics of care. A NICU in a lower-resource region of Africa need not replicate norms of HICs, but focus on essential skills that promote thermoregulation, prompt respiratory support, optimal nutrition, and infection control. Educating hospital management, government officials and ministries of the importance of essential-yet-unavailable NICU drugs (i.e., caffeine, pulmonary surfactant, NICU IV fluids, vitamin D, and levetiracetam) and everyday consumables (i.e., appropriately sized oxygen cannulas and oro/nasogastric tubes, CPAP supplies, quality IV cannulas, tape and syringes) remains as important as any expensive equipment. In the rush to build and equip NICUs in SSA, the additional building blocks of medications, consumables, needed point-of-care tests, properly trained nurses and physicians, appropriate staffing ratios, and a culture of physician-nurse-caregiver teamwork in the NICU is critically important. This is a journey that cannot be achieved with a hurriedly constructed room, donated equipment, and some training workshops. It is a journey of accompaniment that requires patience, advocacy, and hope. It is a lesson in the importance of transforming medical culture to achieve outcomes once considered impossible, but now a reality.

## Data availability statement

The raw data supporting the conclusions of this article will be made available by the authors, without undue reservation.

## Author contributions

SS conceptualized, designed the study, and performed the data analysis. SS, GP, JM, and EM contributed to interpretation. KM, SS, and HS drafted the initial version of the manuscript. GP and EM performed the data collection and were involved in the analysis. JM structured the data and performed the analysis. All authors participated in critical revision of the manuscript for important intellectual content, approved the final manuscript as submitted, and agree to be accountable for all aspects of the work.

## Funding

Charitable care of NICU infants at Arusha Lutheran Medical Center (Tanzania) is partially supported through individual donors, churches, local area businesses, and non-governmental organizations (African Mission Healthcare and Global Health Ministries).

## Conflict of interest

The authors declare that the research was conducted in the absence of any commercial or financial relationships that could be construed as a potential conflict of interest.

## Publisher's note

All claims expressed in this article are solely those of the authors and do not necessarily represent those of their affiliated organizations, or those of the publisher, the editors and the reviewers. Any product that may be evaluated in this article, or claim that may be made by its manufacturer, is not guaranteed or endorsed by the publisher.

## Footnotes

^1^ https://www.eurolyser.com/medical-diagnostics/parameter/crp-test/

^2^ https://nest360.org

^3^ https://www.hatch-tech.org

## References

[1] United Nations Inter-Agency Group for Child Mortality Estimation (UN IGME). Levels and Trends in Child Mortality: Report 2021 Estimates Developed by the United Nations Inter-Agency Group for Child Mortality Estimation. New York, NY: UNICEF (2021).

[2] Perin J Mulick A Yeung D Villavicencio F Lopez G Strong K. Global, regional, and national causes of under-5 mortality in 2000-19: an updated systematic analysis with implications for the Sustainable Development Goals. Lancet Child Adoles Health. (2021) 6:106–15. 10.1016/S2352-4642(21)00311-4 34800370 PMC8786667

[3] United Nations Inter-Agency Group for Child Mortality Estimation (UN IGME). A Neglected Tragedy: The Global Burden of Stillbirths. New York, NY: UNICEF (2020).

[4] Lawn JE Blencowe H Oza S You D Lee A Waiswa P. Every newborn: progress, priorities, and potential beyond survival. Lancet. (2014) 384:189–205. 10.1016/S0140-6736(14)60496-7 24853593

[5] Institute for Health Metrics Evaluation. Global Burden of Deaths 2019. (2019). Available online at: https://vizhub.healthdata.org/gbd-compare/ (accessed May 31, 2022).

[6] PopulationStat. World Statistical Data, Population Data Comparison mid-2022. Available online at: https://populationstat.com/continents/ (accessed July 26, 2022).

[7] Tooke L Ehret DEY Okolo A Dlamini-Nqeketo S Joolay Y Minto'o S. Limited resources restrict the provision of adequate neonatal respiratory care in the countries of Africa. Acta Paediatr. (2022) 111:275–83. 10.1111/apa.16050 34328232

[8] World Health Organization. Survive and Thrive: Transforming Care for Every Small and Sick Newborn. World Health Organization (2019).

[9] Hansen A Magge H Labrecque M Munyaneza RB Nahimana E Nyishime M. The development and implementation of a newborn medicine program in a resource-limited setting. Public Health Action. (2015) 5:17–22. 10.5588/pha.14.0106 26400597 PMC4525367

[10] Magge H Chilengi R Jackson EF Wagenaar BH Kante AM. Tackling the hard problems: implementation experience and lessons learned in newborn health from the African Health Initiative. BMC Health Serv Res. (2017) 17:829. 10.1186/s12913-017-2659-4 29297352 PMC5763287

[11] Tuyisenge D Byiringiro S Manirakiza ML Mutsinzi RG Nshimyiryo A Nyishime M. Quality improvement strategies to improve inpatient management of small and sick newborns across All Babies Count supported hospitals in rural Rwanda. BMC Pediatr. (2021) 21:89. 10.1186/s12887-021-02544-z 33607961 PMC7893907

[12] Our World in Data. Medical Doctors Per 1,000 People. (2017). Available online at: https://ourworldindata.org/grapher/physicians-per-1000-people?tab=chart&country=Sub-Saharan+Africa~Sub-Saharan+Africa+%28IDA+%26+IBRD%29~Sub-Saharan+Africa+%28excluding+high+income%29 (accessed May 28, 2022).

[13] Anyangwe SCE Mtonga C. Inequities in the global health workforce: the greatest impediment to health in sub-Saharan Africa. Int J Environ Res Pu. (2007) 4:93–100. 10.3390/ijerph2007040002 17617671 PMC3728573

[14] Veazie S Peterson K Bourne D. Evidence Brief: Implementation of High Reliability Organization Principles. Washington, DC: Department of Veterans Affairs (2019). 31233295

[15] O'Leary KJ Wayne DB Haviley C Slade ME Lee J Williams V. Improving teamwork: impact of structured interdisciplinary rounds on a medical teaching unit. J Gen Intern Med. (2010) 25:826–32. 10.1007/s11606-010-1345-6 20386996 PMC2896605

[16] Bynum WE Varpio L Lagoo J Teunissen PW. I'm unworthy of being in this space: the origins of shame in medical students. Med Educ. (2020) 5:14354. 10.1111/medu.14354 32790934

[17] Shah BJ Portnoy B Chang D Napp M. Just culture for medical students: understanding response to providers in adverse events. MedEdPORTAL. (2021) 17:11167. 10.15766/mep_2374-8265.11167 34277933 PMC8266940

[18] Saposnik G Redelmeier D Ruff CC Tobler PN. Cognitive biases associated with medical decisions: a systematic review. BMC Med Inform Decis Mak. (2016) 16:138. 10.1186/s12911-016-0377-1 27809908 PMC5093937

[19] Pisoni GB Gaulis C Suter S Rochat MA Makohliso S Roth-Kleiner M. Ending neonatal deaths from hypothermia in sub-Saharan Africa: call for essential technologies tailored to the context. Front Public Health. (2022) 10:851739. 10.3389/fpubh.2022.851739 35462801 PMC9022947

[20] Richards-Kortum RR. Tools to reduce newborn deaths in Africa. Health Aff. (2017) 36:2019–22. 10.1377/hlthaff.2017.1141 29137526

[21] Lunze K Bloom DE Jamison DT Hamer DH. The global burden of neonatal hypothermia: systematic review of a major challenge for newborn survival. BMC Med. (2013) 11:24. 10.1186/1741-7015-11-24 23369256 PMC3606398

[22] Fitzgerald FC Zingg W Chimhini G Chimhuya S Wittmann S Brotherton H. The impact of interventions to prevent neonatal healthcare-associated infections in low-and middle-income countries: a systematic review. Pediatr Infect Dis J. (2022) 41:S26. 10.1097/INF.0000000000003320 35134037 PMC8815829

[23] Olusanya BO Kaplan M Hansen TWR. Neonatal hyperbilirubinaemia: a global perspective. Lancet Child Adolesc Heal. (2018) 2:610–20. 10.1016/S2352-4642(18)30139-1 30119720

[24] Thukral A Sankar MJ Chandrasekaran A Agarwal R Paul VK. Efficacy and safety of CPAP in low- and middle-income countries. J Perinatol. (2016) 36:S21–8. 10.1038/jp.2016.29 27109089 PMC4848740

[25] Guthrie SO Fort P Roberts KD. Surfactant administration through laryngeal or supraglottic airways. Neoreviews. (2021) 22:e673–88. 10.1542/neo.22-10-e673 34599065

[26] WHO Immediate KMC Study Group. Immediate “kangaroo mother caree and survival of infants with low birth weight. N Engl J Med. (2021) 384:2028–38. 10.1056/NEJMoa2026486 34038632 PMC8108485

[27] Hofer N Zacharias E Müller W Resch B. An update on the use of C-reactive protein in early-onset neonatal sepsis: current insights and new tasks. Neonatology. (2012) 102:25–36. 10.1159/000336629 22507868

[28] Thayyil S Pant S Montaldo P Shukla D Oliveira V Ivain P. Hypothermia for moderate or severe neonatal encephalopathy in low-income and middle-income countries (HELIX): a randomised controlled trial in India, Sri Lanka, and Bangladesh. The Lancet Global Health. (2021) 9:e1273–85. 10.1016/S2214-109X(21)00264-3 34358491 PMC8371331

[29] Marks IH Thomas H Bakhet M Fitzgerald E. Medical equipment donation in low-resource settings: a review of the literature and guidelines for surgery and anaesthesia in low-income and middle-income countries. BMJ Global Health. (2019) 4:e001785. 10.1136/bmjgh-2019-001785 31637029 PMC6768372

[30] Schwartz L Lockwood D. Why It's so Hard for a Hospital in Tanzania to Fix Broken Incubators. New York, NY: Rest of World (2021).

[31] Medical Devices: Managing the Mismatch: An Outcome of the Priority Medical Devices Project. Geneva: World Health Organization (2010).

[32] Swanson SJ Martinez KK Stenzel LO Giesbrecht L. Every Breath Counts: Manual of Neonatal Care & Drug Doses. Arusha: Arusha Lutheran Medical Centre (2021).

